# Molecular Composition of Organic Peroxides in Secondary Organic Aerosols Revealed by Peroxide-Iodide Reactivity

**DOI:** 10.1021/acs.est.5c03241

**Published:** 2025-08-07

**Authors:** Kangwei Li, Zhensen Zheng, Julian Resch, Jialiang Ma, Armin Hansel, Markus Kalberer

**Affiliations:** † Department of Environmental Sciences, 27209 University of Basel, Basel 4056, Switzerland; ‡ Institute of Ion Physics and Applied Physics, 27255 University of Innsbruck, Innsbruck 6020, Austria; § IONICON Analytik GmbH, Innsbruck 6020, Austria; ∥ Institute for Atmospheric and Environmental Sciences, 9173 Goethe-University Frankfurt, Frankfurt am Main 60438, Germany

**Keywords:** organic peroxide, molecular composition, secondary organic aerosol, LC-HRMS, iodometry, non-targeted analysis

## Abstract

Organic peroxides are recognized as major contributors to the toxicity and adverse health effects of secondary organic aerosols (SOA). However, their molecular composition and chemical properties in SOA remain largely unexplored. Here we develop a novel analytical strategy for the molecular characterization of organic peroxides in α-pinene SOA, combining iodometry kinetic experiments with liquid chromatography–high-resolution mass spectrometry. Using non-targeted analysis, we identify over 300 organic peroxides in α-pinene SOA, showing a wide range of reactivities with iodide, spanning 4 orders of magnitude. The structures of 12 organic peroxides derived from stabilized Criegee intermediates are further proposed and discussed. Our findings present a reliable, standard-free methodology for identifying previously uncharacterized organic peroxides in SOA at the molecular level, offering new avenues for investigating formation pathways and health impacts of this large, diverse, yet rarely explored compound class. Additionally, we propose peroxide-iodide reactivity as a novel metric for future predictions of oxidative potential—a key indicator of toxicity—and for elucidating the structure of individual organic peroxides in SOA.

## Graphical abstract

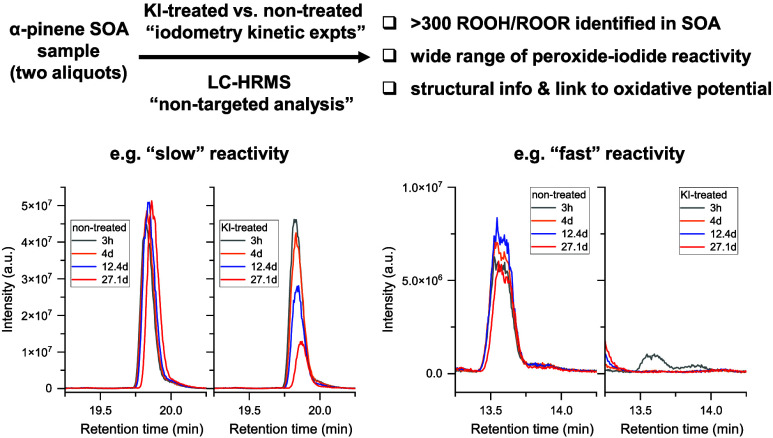

## Introduction

Atmospheric aerosol particles have a great impact on air quality, climate change, and human health.^1^ They are found everywhere in the Earth’s atmosphere and are present in varying composition and concentrations from remote oceanic regions and pristine wilderness areas to heavily industrialized cities. Among different aerosol components, organic aerosols are a significant component contributing 20–70% of the total aerosol mass.^2,3^ Secondary organic aerosols (SOA) are typically formed through (photo)­oxidation of volatile organic compounds (VOCs) in the atmosphere, leading to the formation of less volatile products that condense onto existing particles or nucleate to form new particles.^4^ SOA consists of thousands of different organic compounds, ranging from simple molecules to highly oxidized and multifunctional species. It has been postulated that organic peroxides (ROOH and ROOR, where R represents an organic group), a significant compound class in SOA, may play a pivotal role in the toxicity and related health effects of aerosols.^5^ This is primarily due to their reactive and oxidizing properties, where peroxides are also considered as a compound class contributing to the so-called reactive oxygen species (ROS). Despite the significance of organic peroxides in atmospheric chemistry and human health, the analytical identification and quantification of these compounds in atmospheric aerosols remains highly challenging.^6^

Iodometry is a well-established titration method for quantifying total peroxides because iodide is known to selectively react with peroxides.^5^ Based on such iodometry UV–vis measurements, it has been reported that organic peroxides substantially contribute to SOA mass, though there is still a very large uncertainty regarding the mass fraction of organic peroxides and their molecular-level understanding in SOA.^5^ First, a significant number of organic peroxides present in SOA remain poorly identified and characterized. This is mainly due to the chemical complexity of SOA molecular composition with diverse structures, e.g., co-existence of many peroxide and non-peroxide isomers. In particular, only about ten organic peroxide standards are commercially available, while most of them are not atmospherically relevant, in sharp contrast to a large speciation of organic peroxides present in SOA. Second, the atmospheric formation and transformation pathways of organic peroxides are highly complex.^7^ For example, numerous pathways exist by which organic peroxides can be formed, either in the gas phase or particle phase, or partitioned between the two phases. In addition, organic peroxides are reactive components which can oxidize other compounds,^8^ or undergo degradation due to the labile property of the −O–O– functional group.^9^

Therefore, there is a need of reliable and standard-free analytical method for molecular-level identification of organic peroxide in SOA, which would allow further investigation into their formation chemistry. Recently, Zhao et al.^10^ developed a novel analytical method for molecular identification of organic peroxides in SOA, which combines iodometry and liquid chromatography coupled to high-resolution mass spectrometry (LC-HRMS). Briefly, one aliquot of SOA extract was untreated, while another aliquot was reacted with concentrated KI for several hours, before the two samples were injected into LC-HRMS. Organic peroxides were identified if a specific chromatographic peak disappeared or was largely reduced in the KI-treated sample. Another study^11^ further employed this method and 75 isomer-resolved organic peroxides were identified in α-pinene SOA. These previous studies indeed show iodometry-assisted LC-HRMS as a promising method for molecular identification of organic peroxides in SOA. However, the kinetics between iodide and organic peroxides should be fundamentally determined by the structure of individual peroxides. For instance, a previous study^12^ revealed a large reactivity difference of H_2_O_2_ and peracetic acid with iodide respectively, while iodometric measurements by Banerjee and Budlke^13^ showed very low sensitivity to tertiary dialkyl peroxides such as *tert*-butyl peroxide and dicumyl peroxide. Another analogous example is the hydrolysis of peroxides, although this is a very different type of reaction compared with iodometry. As explored in our recent study,^14^ a number of α-acyloxyalkyl hydroperoxides (AAHPs, a subclass of organic peroxide) with known structures were synthesized and their hydrolysis kinetics were experimentally determined to be very compound-dependent. Given the very large number of structures as well as diverse formation pathways of organic peroxides in SOA, we hypothesize that the peroxide-iodide reaction kinetics could also be compound- and structure-dependent, which is the main motivation of this study.

In this study, we first characterized the kinetics between iodide and individual peroxide standards that are commercially available, which allows us to recognize the range and importance of peroxide-iodide reactivity. Then, we performed iodometry kinetic experiments of α-pinene SOA based on iodometry-assisted LC-HRMS and non-targeted analysis, where more than 300 organic peroxides were unambiguously identified with precise formula information, and extended structure information was further revealed by peroxide-iodide reactivity. Our results significantly expand the molecular-level identification and understanding of organic peroxides in SOA, which represents a fundamental step and prerequisite for future investigation of their formation chemistry, atmospheric transformation, and health impact.

## Materials and Methods

### Chemicals

All 11 peroxide standards were bought from Sigma-Aldrich, including hydrogen peroxide (H_2_O_2_, 30%), peracetic acid (PAA, 36–40%), 3-chloroperbenzoic acid (3-CA, 77%), benzoyl peroxide (BP, with 25% H_2_O), *tert*-butyl hydroperoxide (t-BH, 70%), cumene hydroperoxide (CH, 80%), *tert*-butyl peroxybenzoate (t-BPB, 95%), *tert*-butyl peracetate (t-BPA, 50%), *tert*-butyl peroxide (t-BP, 98%), dicumyl peroxide (DP, 98%), and 2-butanone peroxide (2-BP, 32%). Additional chemicals used in this study include α-pinene (98%, Sigma-Aldrich), potassium iodide (KI, 99.5%, Sigma-Aldrich), acetonitrile (ACN, Optima LC/MS grade, Fisher Scientific), methanol (Optima LC/MS grade, Fisher Scientific), formic acid (Optima LC/MS grade, Fisher Scientific), acetic acid (Optima LC/MS grade, Fisher Scientific), camphorsulfonic acid (98%, Sigma-Aldrich), cyclohexane (99%, Sigma-Aldrich), and water (Optima LC/MS grade, Fisher Scientific).

### Laboratory-Generated SOA and Filter Extraction

Ten different α-pinene SOA samples were collected from flowtube experiments by mixing α-pinene vapor (estimated in several ppm level) with O_3_ in the dark under various conditions. The formed SOA particles were collected onto 47 mm PTFE membrane filters (0.2 μm pore size, Whatman) with a mass loading of ∼ 200 μg. Figure S1 shows the flowtube setup, which incorporates a recently developed instrument, the organic coating unit,^15^ for the consistent generation of VOC vapor prior to its mixing with ozone. These ten flowtube experiments were conducted under a wide range of conditions, including variations in initial ozone concentrations (0.5, 3.5, and 25 ppm), relative humidity (<5%, 40% and 80%), and with or without the addition of cyclohexane as an OH scavenger. After collection, 20 μL of 5 μM camphorsulfonic acid was added on one-fourth of the filter as an internal standard, and then immediately extracted with acetonitrile (ACN). The extracts were then concentrated to complete dryness (Eppendorf Concentrator plus, Fisher Scientific) and reconstituted with 150 μL of ACN before being injected into LC-HRMS. We intentionally use ACN as the extraction solvent to minimize other unwanted decomposition processes such as hydrolysis, as good stability of α-acyloxyalkyl hydroperoxides (AAHPs) in ACN has been observed in our recent study.^14^ The above extraction and preconcentration process is conducted at room temperature. For the SOA iodometry kinetic experiments, ten extracts from ten different α-pinene SOA samples were combined, which should be highly representative as it reflects many different α-pinene SOA generating conditions on average.

### Synthesis of SCI-Derived Organic Peroxides from Liquid-Phase Ozonolysis Experiments

The ozonolysis of alkenes generates a type of reactive intermediates, stabilized Criegee intermediates (SCIs), which can further undergo various bimolecular reactions with water, alcohols, aldehydes, and carboxylic acids to form different SCI-derived organic peroxides, including α-substituted hydroperoxides and secondary ozonides (SOZ). We used an impinger to synthesize a number of SCI-derived peroxides via liquid-phase ozonolysis. Briefly, high concentration of O_3_ (∼500 ppm in clean air, 100 mL min^–1^) was bubbled through a 10 mL ACN solution containing 1 mM α-pinene. O_3_ reacted in solution with α-pinene resulted in the formation of SCIs, which were rapidly scavenged by the other co-produced oxidation products and lead to the formation of SCI-derived organic peroxides. The oxidized compounds produced during the liquid-phase ozonolysis of α-pinene include various carboxylic acids, carbonyls, and other species, and some of them are also expected to be formed during gas-phase ozonolysis of α-pinene. At each time interval, 75 μL of solution was taken from the impinger for LC-HRMS injection, which allows to track the temporal trends of individual compounds. The temporal trend of these SCI-derived organic peroxides should follow a unique pattern with increases in the beginning and then reaching a plateau after ca. 5 min, a critical timing when alkenes are fully consumed and SCIs are no longer available in our condition. Such a unique temporal pattern was first shown in our recent study where we synthesized a number of AAHPs,^14^ and here we extend this idea to synthesize a number of SCI-derived peroxides. Some of these SCI-derived peroxides are expected to be found in α-pinene SOA samples, and their structures can be further inferred based on some known formation pathways.

### Liquid Chromatography Coupled to High-Resolution Mass Spectrometry (LC-HRMS)

LC-HRMS consists of an ultraperformance liquid chromatography unit (ACQUITY UPLC I-Class, Waters) coupled with a high-resolution mass spectrometer (HRMS, Orbitrap Q Exactive Plus, Thermo Scientific). The detailed parameters for liquid chromatography and mass spectrometry are similar to that described in our recent study,^14^ which has been summarized in Table S1. The mass spectrometer was calibrated daily for positive and negative modes using Thermo Scientific Pierce Ion Calibration Solution (Fisher Scientific), while the data presented in this study is mainly from the positive mode. In addition, an HPLC Gradient System Diagnostics Mix (Sigma-Aldrich) containing five compounds was injected daily to monitor the stability of the signal intensity and retention time. The extracted ion chromatograms for selected ions were exported using the Xcalibur 2.2 software (Thermo Scientific) with a mass tolerance of 10 ppm.

### Iodometry Kinetic Experiments Using UV–vis and LC-HRMS

In the first part of iodometry kinetic experiments, the 11 peroxide standards (all commercially available) were reacted with excess iodide. We monitor the temporal pattern of I_3_^–^ formation for each peroxide standard using a UV–Vis spectrometer (LAMBDA 365, PerkinElmer), allowing us to determine their peroxide-iodide reactivity individually. The peroxide standards were prepared and diluted in ACN solution at ∼ 50 μM. Then, 1.8 mL peroxide standard (∼50 μM in ACN) was mixed with 0.1 mL acetic acid (600 mM in ACN) in a vial, followed by the addition of 0.1 mL KI (400 mM in H_2_O). This initiates the iodometry reaction in a 2 mL solution containing ∼ 45 μM peroxide standard, 30 mM acetic acid and 20 mM KI. Formation of I_3_^–^ was regularly measured by UV–vis over a time scale ranging from minutes up to 4 weeks. The addition of acetic acid creates an acidic condition, which provides enough protons for the iodometry reaction to proceed. The solution was stored in a closed vial at room temperature (∼20 °C).

The second part of iodometry kinetic experiments using SOA samples were performed in a similar way as for the peroxide standards described above, where they were regularly measured by LC-HRMS instead of UV–vis. Specifically, 40 μL was taken from each α-pinene SOA extract (150 μL initial volume) and 10 extracts were combined as 400 μL. One aliquot (180 μL) from the combined extract was mixed with 10 μL acetic acid (600 mM in ACN) in a vial, followed by the addition of 10 μL KI (400 mM in H_2_O) to start the iodometry reaction; another 180 μL aliquot was treated in a same way by adding 10 μL acetic acid (600 mM in ACN) and 10 μL H_2_O, instead of KI. As shown in Table S2, these two SOA samples are defined as “KI-treated” and “non-treated”, respectively, which were repeatedly injected into LC-HRMS for 4 weeks at the following time points after iodometry: 3 h, 0.8, 2.1, 4.0, 7.1, 9.0, 12.4, 19.3, and 27.1 days. Note that we directed the LC eluting flow into waste for the first 3 min of the whole 30 min method to minimize possible contamination of KI entering into the HRMS. These KI-treated and non-treated SOA samples were stored in an autosampler at 8 °C during the 4 weeks of repeated analyses, with a closed cap to avoid evaporation.

### Non-targeted Analysis of LC-HRMS Dataset and Organic Peroxide Identification

The LC-HRMS raw data files were converted to mzML format using the ProteoWizard (MSConvert, version 3) software and were subsequently analyzed by MZmine 4.2.0.^16,17^ An optimized workflow includes mass detection, chromatogram building and deconvolution, ^13^C isotope filtering, retention time alignment, feature grouping and formula prediction. The non-targeted analysis workflow and detailed parameters used in this study for MZmine are summarized in a batch file as Supporting Information, which allows for reproducible LC-HRMS data processing by other MZmine users. The organic peroxide identification is based on an in-house data processing tool with graphical user interface to allow for efficient data filtering and visualization, which are described in Text S1 and Figure S2.

## Results and Discussion

### Compound-Dependent Reactivity of Peroxide Standards with Iodide

The rate constants for peroxides reacting with iodide are mostly unknown, and therefore we first characterize the reaction kinetics of individual peroxide standards with iodide. Eleven peroxide standards are selected, including 1 inorganic peroxide (H_2_O_2_) and 10 organic peroxides with diverse structures, as shown in Figure 1. These peroxides are selected due to their commercial availability, though most of them are not atmospherically relevant. It is well-known that peroxides can oxidize iodide and finally lead to the formation of I_3_^–^, which can be easily identified by UV–vis measurement. As mentioned in Method Section, we performed iodometry kinetic experiments in ACN-dominated solutions (95% ACN + 5% H_2_O) containing ∼ 45 μM peroxide standard, 30 mM acetic acid and 20 mM KI at room temperature. We monitored the I_3_^–^ absorption as a function of time, which allows for a relative comparison of the peroxide-iodide reactivity (*k*_1st_) among those peroxide standards.

**Figure 1:**
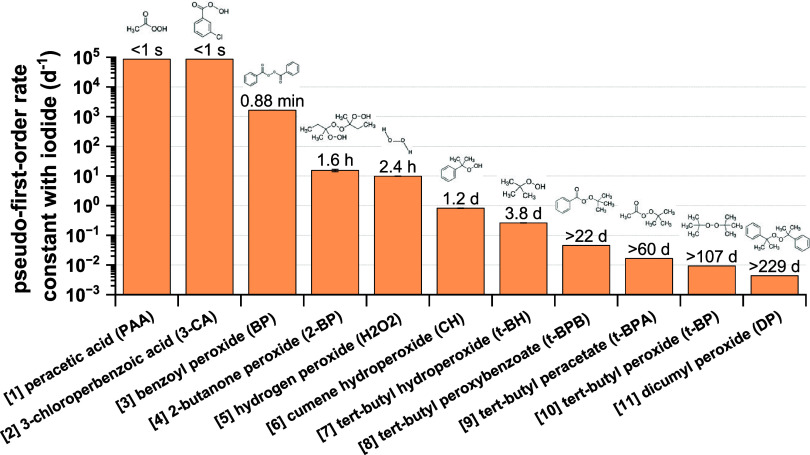
Summary of peroxide-iodide pseudo-first-order rate constants (*k*_1st_) for the 11 commercially available peroxides with diverse structures. The reactivity was fitted or estimated from iodometry kinetic experiments based on UV–vis data, which are described and summarized in the Text S2, Figure S3 and Table S3. The rate constant (8.64 × 10^4^ d^–1^) was estimated as a lower limit for [1] PAA and [2] 3-CA according to their *e*-folding lifetime (<1s), and the rate constants for [8] t-BPB, [9] t-BPA, [10] t-BP and [11] DP were estimated as upper limit. The *e*-folding lifetime (τ_1/e_) for each peroxide is labeled above each bar.

I_3_^–^ has an absorption peak typically at 350–353 nm in water,^18,19^ and we noticed that the I_3_^–^ absorption peak shifts to 361 nm in ACN-dominate solvent (i.e., 95% ACN + 5% H_2_O in this study), which has been described in details in the Text S2. As shown in Figure S3, we observed large differences of I_3_^–^ temporal profiles among these peroxide standards, which illustrates the large variation of I_3_^–^ formation rate between individual peroxide and iodide. For instance, PAA and 3-CA were fully titrated to I_3_^–^ within less than 1 s, while the I_3_^–^ formation of some peroxides (e.g., t-BPA, t-BPB, t-BP and DP) does not reach a plateau even after more than 2 weeks.

Since I^–^ is in large excess, the *k*_1st_ values for these selected peroxides with iodide can be determined or estimated, as described in Text S2. Among all peroxide standards, peracids such as PAA and 3-CA show highest reactivity with iodide, which is not surprising. It is known that the oxidation rate of iodide by PAA is much faster than that by H_2_O_2_.^12^ In addition to its presence in the atmosphere,^20^ PAA is also widely used in other fields due to its highly oxidative ability, such as advanced oxidation technology in wastewater treatment.^21^ To the best of our knowledge, the available peroxide-iodide kinetic data in the literature are limited to PAA and H_2_O_2_ with the reported aqueous rate constants differing about 4–5 orders of magnitude (i.e., 4.22 × 10^2^ M^–1^ s^–1^ for PAA^22^ and 9.50 × 10^–3^ M^–1^ s^–1^ for H_2_O_2_ ^23^ at similar pH = 4.3 for both compounds), which quantitatively agrees with our observation.

As summarized in Figure 1 and Table S3, these peroxide standards are classified into five subgroups, with the *k*_1st_ values generally following the order: R­(C=O)­OOH (10^5^ d^–1^) > R­(C=O)­OO­(C=O)­R (10^3^–10^4^ d^–1^) > ROOH ≈ H_2_O_2_ (10^–1^–10 d^–1^) > R­(C=O)­OOR (10^–2^–10^–1^ d^–1^) > ROOR (10^–3^–10^–2^ d^–1^). This compound- and structure-dependent reactivity of peroxide with iodide can be explained by the specific position of the peroxide functional group. For example, the peroxide bond becomes more reactive when neighboring a hydrogen atom (i.e., hydroperoxides), rather than an organic group. In addition, the presence of a carbonyl group next to the peroxide functional group also increases the reactivity of organic peroxides. The measurement of *k*_1st_ values with such a wide range indicates an important metric, which directly links to the peroxide structures and provides substructural information, as explored in the next sections.

### Identification of Organic Peroxides in SOA through Non-targeted Analysis

Considering the wide range of rate constants of organic peroxides with iodide determined above (Figure 1), it is reasonable to assume that the kinetics of organic peroxides in SOA with iodide will also be very compound-dependent and vary over many orders of magnitude. We test this idea by performing SOA iodometry kinetic experiments, where a number of α-pinene SOA samples were collected under different experimental conditions and their combined extracts were divided into two aliquots. One aliquot was treated with KI and the other was not treated, and both sets were injected repeatedly into LC-HRMS over a time period of 4 weeks (see Method Section). This time-dependent iodometry-assisted LC-HRMS is a further development of the method initially proposed by Zhao et al.^10^

We performed non-targeted analysis for the whole LC-HRMS dataset to efficiently visualize the data and identify organic peroxides (see Method Section, Text S1 and Figure S2). Figure 2 shows several representative examples of chromatographic peaks of the identified organic peroxides, including their time-dependent extracted ion chromatograms (EICs) and KI-treated/non-treated ratios. This is in sharp contrast to a number of non-peroxides with known structures (e.g., carboxylic acid, carbonyl, ester, etc.) as shown in Figure S4, which do not decay over 4 weeks and are overall stable in both KI-treated and non-treated conditions. This highlights the reliability and selectivity of identifying organic peroxides by this method that enables to distinguish them from other non-peroxide compound classes. Such type of temporal pattern would allow us to identify individual organic peroxide unambiguously, especially when multiple adducts are found. For example, each suggested formula is constrained by at least two adducts and one ^13^C isotope pattern, and some adducts are matched to H_2_O neutral loss (e.g., C_20_H_32_O_6_ and C_12_H_20_O_5_ as shown in Figures 2C,2D), which are all within a retention time (RT) tolerance of 0.1 min and a mass accuracy of 5 ppm. In-source fragmentation was also considered.^24,25^ For example, the compound C_10_H_16_O_2_ has the same RT as the much larger compound C_20_H_20_O_5_ both eluting at 20.7 min (Figure 2A), illustrating that C_10_H_16_O_2_ is a fragment resulting from in-source fragmentation of C_20_H_20_O_5_. It is generally accepted that non-targeted analysis based on LC-HRMS could generate chemical profiles with different levels of confidence, such as chemical formula prediction without structure (confidence level 4), with tentatively assigned structure (confidence level 3), with more accurate structure (confidence level 2), and with structure confirmed by reference standard or NMR analysis (confidence level 1), according to Schymanski et al.^26^ All these above criteria give a level 4 identification confidence for our peroxide formula assignment.

**Figure 2:**
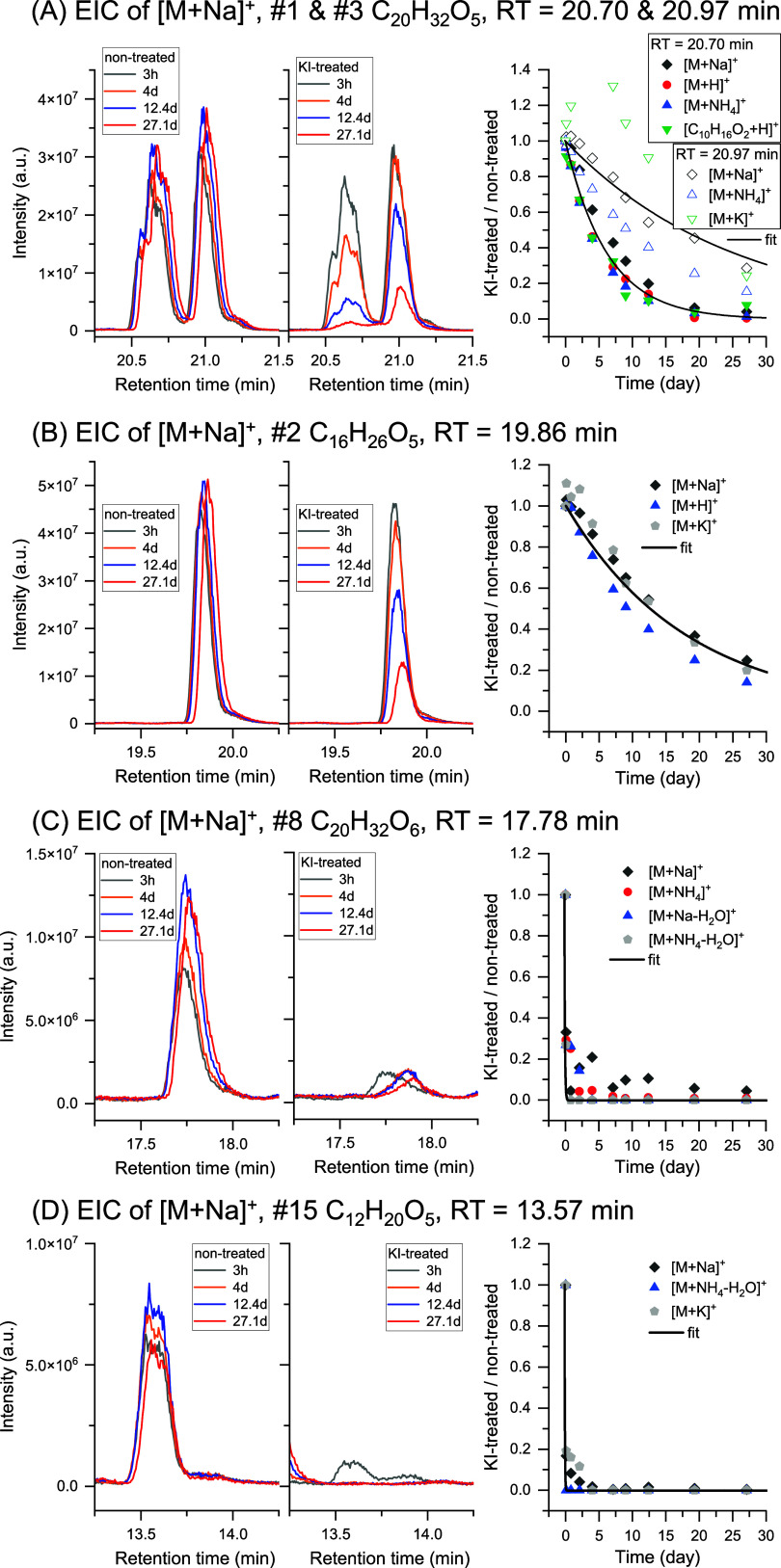
Five representative organic peroxides identified in α-pinene SOA through time-dependent iodometry-assisted LC-HRMS. For each organic peroxide (#1, #2, #3, #8, #15, the rank number is sorted by intensity and summarized in Table S4), time-dependent extracted ion chromatograms (EICs) from KI-treated and non-treated dataset are shown, as well as temporal trends of KI-treated/non-treated ratios (right panels). The exponential fit with equation *y* = exp­(−*k*_1st_ *t*), following pseudo-first-order kinetics, applies to the merged data of multiple adducts from the same compound.

We use the exponential decay profile of KI-treated/non-treated ratios to determine *k*_1st_ values, i.e., the pseudo-first-order reaction rate constants between organic peroxide and iodide, which rules out any other non-iodometry effects such as daily variation of instrument sensitivity, sample evaporation, etc. As shown in Figure 2, some organic peroxides such as C_12_H_20_O_5_ (RT = 13.57 min) and C_20_H_32_O_6_ (RT = 17.78 min) show fast decay with τ_1/e_ much less than 1 day, while the relatively slow decay profile such as C_16_H_25_O_5_ (RT = 19.86 min) suggests τ_1/e_ of more than 10 days. Such individual decay profiles of KI-treated/non-treated ratio indicate the large reactivity difference among organic peroxides in SOA, and demonstrates that *k*_1st_ values are very compound-dependent as also shown in Figure 1 for the a few selected peroxide standards. This observation is also supported by the differences in reactivity for isomers. As illustrated in Figure 2A, two isomers of C_20_H_32_O_5_ eluting at 20.70 and 20.97 min respectively, show different decay rates (τ_1/e_ = 6.3 and 18.1 days, respectively), which is most likely attributed to their different molecular structures. Note that C_12_H_20_O_5_ (RT = 13.57 min) and C_20_H_32_O_6_ (RT = 17.78 min) are structure known AAHPs, where both of them were previously synthesized and identified in α-pinene SOA in our recent study.^14^ Their successful identification in this study suggests the reliability of the new analytical method, without relying on authentic standards.

A total number of 374 organic peroxides are identified with confidence level 4 in α-pinene SOA, where the top 50 compounds are summarized in Table S4, sorted by their intensity, together with their *k*_1st_ values and τ_1/e_. Figure 3 shows the intensity (averaged from the non-treated dataset) of these organic peroxides identified in α-pinene SOA as a function of their decay rates (*k*_1st_). The 374 organic peroxides are categorized into monomers (C_6_–C_10_, *n* = 30), dimers (C_11_–C_20_, *n* = 209) and higher order oligomers (C_21_–C_30_, *n* = 130 and C_31_–C_40_, *n* = 5). It clearly shows that the intensity of these organic peroxides varies by about 3 orders of magnitude, and their *k*_1st_ values differ by up to 4 orders of magnitude, regardless of monomeric, dimeric or oligomeric structure. Interestingly, the error bar of the intensity reflects the variability of individual peroxide in the non-treated dataset, which overall correlates with peroxide-iodide reactivity. For instance, organic peroxides falling into the “very slow” category tend to be less variable from the intensity, which may imply their stability in aerosols. As shown in the pie chart of Figure 3, more than 65% of organic peroxides have a *k*_1st_ value less than 1 d^–1^ (equivalent to τ_1/e_ longer than 1 day), which contributes more than 75% of the total peroxide intensity. For those 374 organic peroxides, we attempt to correlate *k*_1st_ with their molecular characteristics such as C number, O number, O/C, H/C, RT and molecular weight. As shown in Figure S5, these scatters do not show clear correlations but grouping them in box plots may indicate some correlations, e.g., higher O/C and lower RT generally correlate with higher *k*_1st_. Note that *k*_1st_ values vary widely at each bin, thus we are unable to parameterize or predict *k*_1st_ values based on molecular formula and RT.

**Figure 3:**
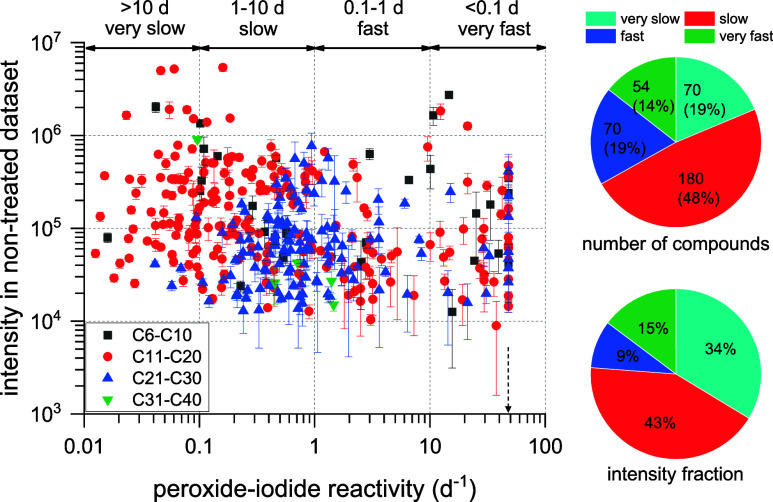
Intensity of 374 organic peroxides identified in α-pinene SOA as a function of their reactivity with iodide (*k*_1st_). The intensity and error bars reflect their average and standard deviation of primary adduct form in the non-treated dataset, including 9 measurements made by LC-HRMS over 4 weeks. When multiple adducts were identified for a given organic peroxide, primary adduct was determined based on the maximum of intensity among each adduct. For the two pie charts, the number and intensity fraction of organic peroxides are divided into the four peroxide-iodide reactivity ranges. A lower limit *k*_1st_ value of 48 d^–1^ (by assuming τ_1/e_ = 0.5 h) shown with a dashed arrow was assumed for some superfast reacting peroxides, as their intensity decayed to zero at 3 h after iodometry and exponential fitting was therefore not possible.

Using this new methodology, the identification ability of organic peroxides in SOA is greatly expanded based on their reaction kinetics with iodide. In particular, some structures and formation chemistry of organic peroxides can be qualitatively categorized based on their *k*_1st_ values. For example, pathways leading to ROOH formation are likely grouped into the “fast” and “very fast” categories, such as SCI-derived α-substituted hydroperoxides (e.g., AAHP), reactions between RO_2_ and HO_2_, and especially some autoxidation processes leading to ROOH with multiple -OOH functional groups.^27^ On the other hand, processes such as biomolecular reactions between RO_2_ and RO_2_, SCI and carbonyls (forming secondary ozonides) can lead to ROOR formation, which are likely being grouped into “slow” and “very slow” category. If these “very slow” peroxides are assumed to be ROOR (e.g., from SCI + carbonyl, RO_2_ + RO_2_, etc.), they may represent an important fraction of the total peroxides. This contrasts with <3% ROOR in total peroxides of α-pinene SOA determined by Alba-Elena et al.^28^ The different α-pinene SOA generation conditions between the two studies may contribute to this difference. We also would like to emphasize that the peroxide-iodide reactivity method applied here cannot directly distinguish between ROOH and ROOR unambiguously, especially considering that ROOR species in SOA may have very different structures and properties compared with those commercial peroxides tested here, mainly containing *tert*-butyl groups. Nevertheless, the peroxide-iodide reactivity provides a key information that holds great promise to further differentiate the numerous formation pathways leading to organic peroxides.

### Proposed Structures of SCI-Derived Organic Peroxides

The above results illustrate that peroxide-iodide reactivity is strongly related to the structure of peroxides, and thus may be helpful toward a structure elucidation. Structural characterization of organic peroxides in SOA can provide molecular-level understanding of their formation chemistry. However, accurate structure assignments for all these identified organic peroxides in SOA are not possible. Nevertheless, we may still infer some structures based on existing knowledge of organic peroxide formation chemistry.

Previous studies suggest that reactions of stabilized Criegee intermediates (SCIs) forming dimers is a major channel leading to the formation of organic peroxides, such as α-hydroxylalkyl hydroperoxides (HAHPs), α-alkoxyalkyl hydroperoxides (AHPs), AAHPs and secondary ozonides (SOZs).^5^ Here we synthesize a number of SCI-derived organic peroxides through liquid-phase ozonolysis experiments. As described in Method Section, the temporal trend of the formation of these SCI-derived organic peroxides should all follow a common pattern that increases at the beginning and then reaches a plateau after ca. 5 min under our liquid-phase ozonolysis conditions,^14^ when the alkene (α-pinene in this case) is fully consumed and SCIs are no longer available. Such temporal pattern allows us to identify SCI-derived peroxides. It is expected that both liquid-phase and gas-phase ozonolysis of α-pinene share some similar oxidation products, and therefore some SCI-derived peroxides synthesized in the liquid-phase ozonolysis experiments are expected to be found in the α-pinene SOA samples.

As summarized in Table 1, 12 out of 374 organic peroxides found in α-pinene SOA are confirmed as SCI-derived peroxides, which are based on (i) their unique temporal trends in liquid-phase ozonolysis experiments (see Figure S6) and (ii) their identification as organic peroxide in SOA iodometry kinetic experiments (see Figure S7). The formulas and suggested structures of their monomer partners are also summarized in Table 1, which are derived by subtracting the C_10_ SCI monomer (e.g., C_10_H_16_O_3_) of α-pinene. Most of these monomers have been previously identified in the literature as oxidation products from α-pinene.^29,30^

**Table 1:**
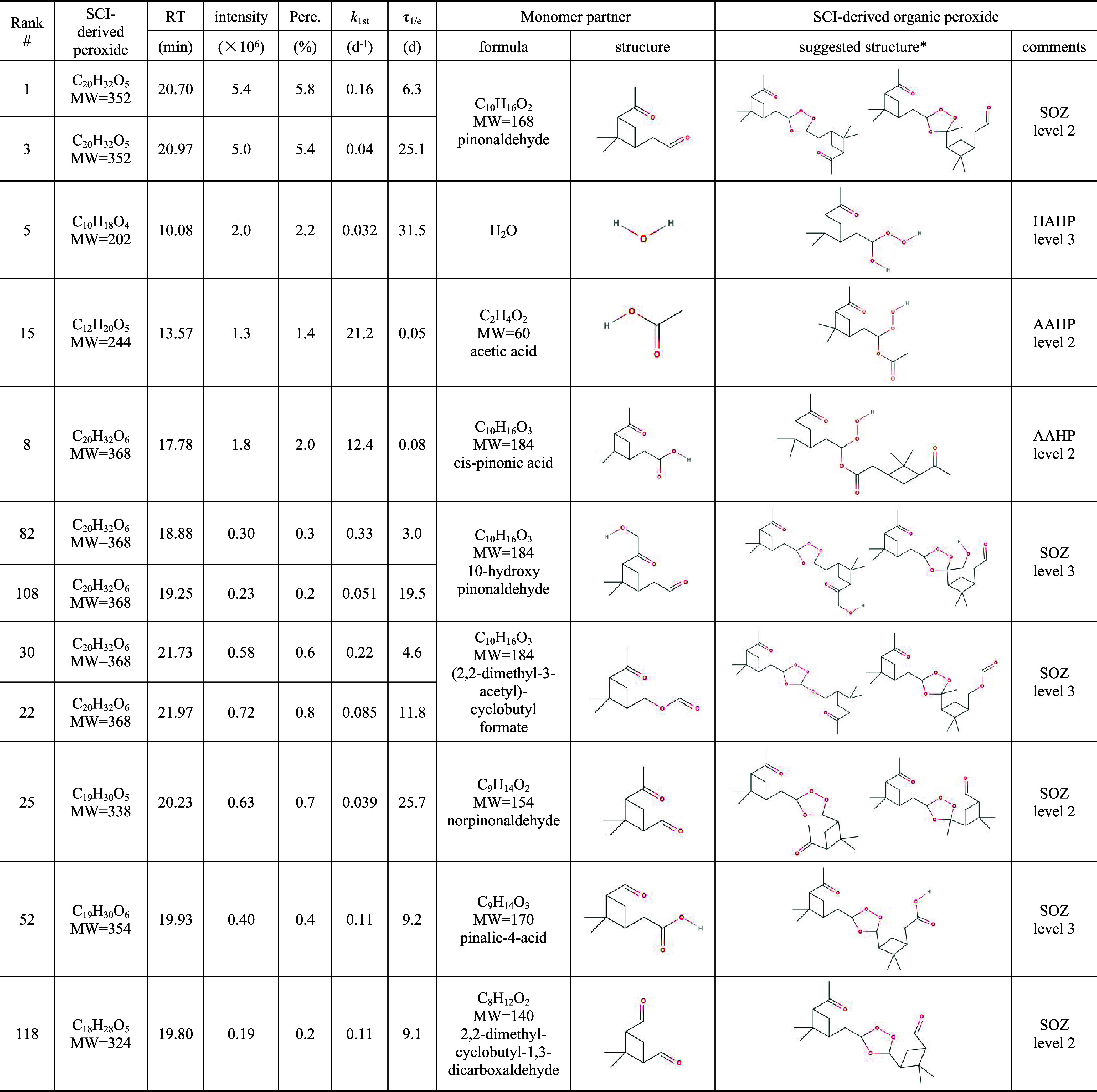
Summary of 12 SCI-Derived Organic Peroxides Identified in α-Pinene SOA

* Note: For these suggested structures of SCI-derived organic peroxides, only one C_10_ SCI form is considered in this table, although four different SCI structures^31^ are known from the ozonolysis of α-pinene and thus four different peroxides are potentially formed. The identification confidence of level 2 refers to no other possible monomer partner based on the best of our knowledge, and level 3 refers to the existence of other possible monomer partners or other uncertainty.^26^

Most of these SCI-derived peroxides are suggested as SOZs, and their *k*_1st_ values (max = 0.33 d^–1^, min = 0.039 d^–1^) further support their structures as SOZs. The two most abundant SOZs (rank number #1 and #3 sorted by intensity as shown in Table S4) contribute more than 10% of total peroxide intensity. Both #1 and #3 are isomers and should have similar structures due to their similar RT of 20.70 and 20.97 min, respectively. They are suggested to be formed from the bimolecular reaction between pinonaldehyde (C_10_H_16_O_2_) and C_10_ SCI at confidence level 2, depending on bonding to C=O or −CHO of pinonaldehyde. Pinonaldehyde is one of the most abundant oxidation product from α-pinene, with reported molar yield (sum of gas and particle phase) of 10–19%.^29^ Similar to pinonaldehyde, norpinonaldehyde (C_9_H_14_O_2_) and 2,2-dimethyl-cyclobutyl-1,3-dicarboxaldehyde (C_8_H_12_O_2_) are suggested as the monomer partners of #25 and #118, which are two SOZs with identification confidence level 2. The elution of these four peroxides follows the order of 19.80 min (#118) < 20.23 min (#25) < 20.70 min (#1) < 20.97 min (#3), which is qualitatively consistent with their increasing the molecular weight (MW) and thus reduced polarity.

As mentioned before, two organic peroxides (#8 and #15) are known AAHPs, as they were previously synthesized in our liquid-phase ozonolysis of α-pinene experiments following the addition of *cis*-pinonic acid and acetic acid, respectively.^14^ Both of the two AAHPs show very fast reactivity (>10 d^–1^) with iodide, consistent with their known structures as ROOH. Note that *cis*-pinonic acid is an example that has both −COOH and C=O functional groups, but the addition of *cis*-pinonic acid during liquid-phase ozonolysis of α-pinene only leads to the formation of AAHP rather than both AAHP and SOZ.^14^ This is qualitatively consistent with a previous finding on kinetic study of C_13_ SCI from ozonolysis of 1-tetradecene, where the reaction of SCI with formaldehyde was found to be much slower than carboxylic acids such as formic acid and heptanoic acid.^32^ This suggests that the carboxyl groups are in general more reactive than carbonyls toward SCI, if the monomer partner has multiple functional groups. For #52 (C_19_H_30_O_6_), the reaction partner of SCI is C_9_H_14_O_3_ (MW = 170), but there are several MW = 170 candidates from the oxidation of α-pinene such as norpinonic acid, pinalic-4-acid and other isomers.^29,30^ Norpinonic acid has both −COOH and C=O functional groups that are similar to *cis*-pinonic acid, which is expected to form AAHP rather than SOZ when reacting with SCI. However, #52 shows a peroxide-iodide reactivity of 0.11 d^–1^, suggesting that it is more likely a SOZ. We tentatively assign this reaction partner as pinalic-4-acid and thus the structure of #52 is suggested at confidence level 3, as we cannot exclude other possible structures of MW = 170.

There are four chromatography peaks with the formula C_20_H_32_O_6_ (#82, #108, #30, #22) that are assigned as SOZs based on their peroxide-iodide reactivity range, which are likely formed in the reaction with two isomers of C_10_H_16_O_3_ (MW = 184). There are also several possible candidates for MW184, such as *cis*-pinonic acid, hydroxy pinonaldehyde or (2,2-dimethyl-3-acetyl)-cyclobutyl formate.^29,30^ *cis*-Pinonic acid is unlikely the correct reaction partner, given that only AAHP (rather than both AAHP and SOZ) was found in our recent study.^14^ 1-Hydroxy pinonaldehyde and 10-hydroxy pinonaldehyde are known oxidation product of pinonaldehyde.^30^ We tentatively assign 10-hydroxy pinonaldehyde rather than (2,2-dimethyl-3-acetyl)-cyclobutyl formate as the reagent forming #82 and #108, which elute at earlier RT (18.88 and 19.25 min) compared with #1 and #3 (20.70 and 20.97 min), consistent with the increased polarity due to the addition of the −OH functional group. However, 1-hydroxy pinonaldehyde could also be a possible candidate. Therefore, the structures of #82 and #108 are suggested at confidence level 3. Similarly, we tentatively assign (2,2-dimethyl-3-acetyl)-cyclobutyl formate as the reaction partner to form #30 and #22, though other unknown structures of MW = 184 may exist. Interestingly, by gathering all information from the three isomeric pairs of SOZs (#1 and #3, #30 and #22, #82 and #108), they show a highly consistent elution order, e.g., the one with a higher *k*_1st_ value always elutes earlier, with a factor of 2–6 difference in τ_1/e_. Among the 12 organic peroxides, #5 (C_10_H_18_O_4_) has the most uncertain structure, and is expected to form in the reaction between C_10_ SCI and H_2_O, leading to the formation of HAHP, a subcategory of hydroperoxide. However, the *k*_1st_ value of #5 (0.032 d^–1^) does suggest it to be a very slowly reactive peroxide, likely belonging to ROOR. Therefore, the structure of #5 is only tentatively assigned with confidence level 3.

Regarding the structures of these 12 SCI-derived organic peroxides in α-pinene SOA, some have been either tentatively or accurately proposed previously such as SOZ from pinonaldehyde,^33^ HAHP from H_2_O,^34^ and AAHPs from *cis*-pinonic acid and acetic acid,^14^ while the remaining peroxides have not been proposed in the literature to the best of our knowledge. We provide a solid methodology to suggest their possible structures by collecting different aspects of information from SOA iodometry kinetics experiments, liquid-phase ozonolysis experiments, and chromatographic elution times and polarity analysis. Some of the structures listed in Table 1 would need further detailed and accurate structural characterization, e.g., via NMR analysis. Nevertheless, the above examples clearly reinforce that the peroxide-iodide reactivity is a useful metric to elucidate the formation pathways and structures of organic peroxides in SOA.

### Atmospheric Implications

Based on our novel time-dependent iodometry-assisted LC-HRMS and non-targeted analysis, we have identified more than 300 organic peroxides in α-pinene SOA, with a wide range of reactivities with iodide spanning over 4 orders of magnitude. Such wide peroxide-iodide reactivity suggests diverse structures. A large fraction (>65%) of organic peroxides show “slow” and “very slow” iodide reactivity with *e*-folding lifetimes longer than 1 day. Some SCI-derived organic peroxides are identified with high confidence in α-pinene SOA with previously known structures (mainly AAHPs) and newly proposed structures (mainly SOZs), which demonstrates that peroxide-iodide reactivity can provide structural information.

We suggest peroxide-iodide reactivity to be considered as a new metric to identify and characterize organic peroxide in future studies for two main reasons. First, the peroxide-iodide reactivity is represented as a chemical property that provides valuable subclass structure information for organic peroxide. MS^2^ and MS^n^ are known to be useful for structure characterization for unknown compounds, which have been applied to SOA studies.^35,36^ However, the long list of structure candidates predicted by MS^2^ (e.g., SIRIUS, Mass Frontier) is still the main challenging issue. Combining peroxide-iodide reactivity with MS^2^ information would allow to narrow down the candidate structures. Second, the peroxide-iodide reactivity metric indicates the oxidizing capability of organic peroxides. As shown in Figure 4, a strong correlation was found between peroxide-iodide reactivity (determined in this study) and oxidative potential (OP) determined with different assays for several peroxide standards from literature data.^37,38^ OP of aerosol particle refers its capability to generate ROS and induce oxidative stress in biological system,^39–41^ which is an emerging health-related metric as a proxy of aerosol toxicity and has been suggested to be one of the possible drivers of the acute health effects of particulate matter.^42–44^ Organic peroxides are known as OP active components in SOA, and such strong correlation among these independent measurements shown in Figure 4 suggests that peroxide-iodide reactivity could be used to estimate or predict OP of individual peroxide in future health assessment studies. However, OP measurements are not the focus of this study, and more experimental evidence is needed to explore this correlation between peroxide-iodide reactivity and peroxide OP and potentially toxicity in more detail.

**Figure 4:**
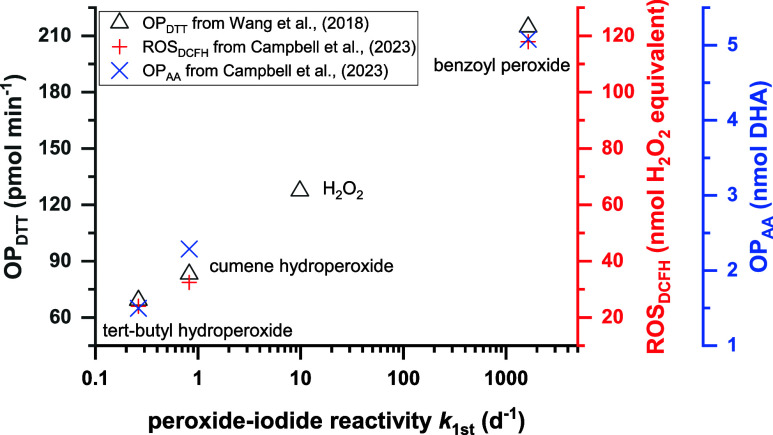
Correlation between peroxide-iodide reactivity (*k*_1st_) and oxidative potential (OP). The OP_DTT_ data for H_2_O_2_ and three organic peroxide standards (all at 120 μM) are from Wang et al.,^37^ which were determined by dithiothreitol (DTT) assay. The ROS_DCFH_ and OP_AA_ data for the three organic peroxide standards (5.55 μM *tert*-butyl hydroperoxide, 3.28 μM cumene hydroperoxide and 2.06 μM benzoyl peroxide) are from Campbell et al.,^38^ using 2,7-dichlorofluoroscein (DCFH) and ascorbic acid (AA) assay, which were normalized to 1 μM peroxide for comparison, assuming that ROS_DCFH_ and OP_AA_ responses with peroxides are linear within a peroxide concentration factor of 2 to 3.

Another potential implication might be linked to Fenton-like reactions involving organic peroxides. It is known that Fe^2+^ reacts with PAA very quickly and its rate constant has been recently determined at 10^4^∼10^5^ M^–1^ s^–1^ under pH = 3–7,^45^ which is 2–3 orders of magnitude higher than that of H_2_O_2_ (∼77 M^–1^ s^–1^).^46^ This difference in reactivity is quite similar to the oxidation of iodide by PAA and H_2_O_2_. Very recently, it has been shown that PAA and 3-CA generate OH radicals very quickly (a so-called OH burst) when mixed with Fe^2+^ in bulk solution, while such an OH burst is not observed with H_2_O_2_ and other organic peroxides such as cumene hydroperoxide, *tert*-butyl hydroperoxide, etc.^47^ This observation leads us to hypothesize that “OH burst” might be positively correlated with peroxide-iodide reactivity. Therefore, our new peroxide-iodide reactivity metric would allow us to identify and prioritize those fast-reacting organic peroxides in SOA, which might be good candidates contributing to the above-mentioned Fenton-like reactions, leading to the formation of OH radicals with high yields. This will help to understand OH sources related to aerosol-cloud chemistry especially in urban and marine environment where iron is an important component.^48,49^

This novel analytical approach based on peroxide-iodide reactivity is represented as a reliable, standard-free methodology that significantly expands the molecular-level identification capability of organic peroxides in SOA, which offers numerous opportunities to further study their detailed formation and transformation chemistry on a molecular level. For instance, while this study focuses on α-pinene SOA, such methodology could be extended to many other SOA systems with different precursors and conditions or ambient samples. In addition, organic peroxides are known to be formed via various pathways such as involving RO_2_, SCI, etc. Based on this new methodology, it is possible to further differentiate the molecular composition of organic peroxides derived from different reaction pathways through well-designed targeted experiments, e.g., by introducing OH, HO_2_, RO_2_ or SCI scavengers. It should be noted that the iodometry kinetic experiments for α-pinene SOA were conducted at 8 °C (e.g., the temperature of the autosampler) and in the presence of 30 mM acetic acid. While maintaining selectivity for peroxides, the peroxide-iodide reactivity developed here could be accelerated by adjusting the solvent acidity or increasing the temperature (e.g., at room temperature). This would allow the whole analytical method to be shortened and therefore not necessarily limited by 4 weeks as presented in this study. Overall, this study highlights the importance of compound- and structure-dependent peroxide-iodide reactivity, and such methodology is expected to improve our molecular understanding of organic peroxides and evaluate their health impact in atmospheric aerosols in future studies.

## Supplementary Material







## Notes

Additional details and summary for identification workflow of organic peroxides in SOA, iodometry kinetic experiments, flowtube setup for SOA generation, LC-HRMS operating conditions, complete list of 374 organic peroxides identified in α-pinene SOA, as well as further results for identification of SCI-derived organic peroxides in α-pinene SOA (PDF)
Dataset of 374 organic peroxides identified in α-pinene SOA (XLSX)
Non-targeted analysis workflow and detailed parameters used in this study for MZmine software 4.2.0 (ZIP)
