# Supplementary material for: Molecular Composition of Organic Peroxides in Secondary Organic Aerosols Revealed by Peroxide-Iodide Reactivity
Source: Environ Sci Technol. 2025 Aug 7;59(32):17126–36. doi: 10.1021/acs.est.5c03241 (PMC12369007; doi:10.1021/acs.est.5c03241)
Supplement: Supplementary file 1 [file es5c03241_si_001.pdf]

Supporting Information for

Molecular composition of organic peroxides in secondary organic aerosols revealed by peroxide-iodide reactivity

Kangwei Li [1*], Zhensen Zheng [2,3], Julian Resch [1], Jialiang Ma [4], Armin Hansel [2], Markus Kalberer [1*]

Affiliations

1. Department of Environmental Sciences, University of Basel, Basel 4056, Switzerland
2. Institute of Ion Physics and Applied Physics, University of Innsbruck, Innsbruck, Austria
3. IONICON Analytik GmbH, 6020 Innsbruck, Austria
4. Institute for Atmospheric and Environmental Sciences, Goethe-University Frankfurt, Frankfurt am Main, 60438, Germany

*Correspondence to: Kangwei Li (kangwei.li@unibas.ch), Markus Kalberer (markus.kalberer@unibas.ch)

This PDF file includes:

Supplementary Text S1 to S2
Figs. S1 to S7
Tables S1 to S4
References (1 to 8)

Other Supplementary Materials for this manuscript include the following:

Data S1: an excel sheet of complete list of 374 organic peroxides identified in α-pinene SOA

Supplementary Text

Text S1: Data processing tool for identification of organic peroxides in SOA

The LC-HRMS dataset was processed following an optimized non-targeted analysis workflow, and the batch file used in MZmine with detailed parameters and settings is attached as supplementary material to allow for reproducible data processing by MZmine users. About 25 000 features were generated by MZmine, where each feature contains a specific *m/z* and retention time (RT). For these features within the same RT window (0.1 min) but also matching ^{13}C isotopes and at least two different adduct forms, they belong to the same compound and therefore are grouped with unique compound ID. With this, nearly 2000 compounds (corresponding to ca. 6000 features) with unique ID and molecular formula were derived and annotated at confidence level 4, while the remaining about 19 000 features that cannot get a confident formula prediction were annotated at confidence level 5, according to Schymanski et al.[1] For organic peroxide identification, we specifically developed an in-house data processing tool (Matlab 2023a, MathWorks) with graphical user interface (GUI), which allows for data visualization and efficient filtering of organic peroxides among these 2000 compounds. The selection process includes the following three steps.

Step 1: Feature that is considered to be an organic peroxide candidate should meet the below robust criteria: (i) should be detected in at least 5 samples across the non-treated dataset (in total 9 samples); (ii) for a given feature, its average intensity in the KI-treated dataset should be less than in the non-treated dataset; (iii) minimum KI-treated/non-treated ratio should be less than 70%; (iv) should have a clear decay profile of KI-treated/non-treated ratio based on fitting parameters; (v) should have a clear decay profile of intensity in the KI-treated dataset based on fitting parameters; (vi) not found in blank samples. After applying these robust selection rules, more than 90% of the 25000 features were removed.

Step 2: The remaining features were then carefully checked individually. This is achieved by a GUI module to visualize the time series of feature intensity in the KI-treated and non-treated dataset, and also the time series of the KI-treated/non-treated ratio. Some features with unrealistic temporal trends were removed manually during this step and only features with reasonable temporal trends were considered for Step 3.

Step 3: As mentioned before, features that have multiple adducts but belong to the same compound are grouped to a unique compound ID, including neutral formula and RT. In this step, we firstly generate a list of all compound IDs that are considered to be organic peroxide candidates, where each contains at least one feature being annotated as an organic peroxide candidate in step 2. For each compound ID, we merge different adducts and check their temporal trend of the KI-treated/non-treated ratio, fitted assuming pseudo-first order kinetics with equation $y = \exp(-k_{1st}t)$ as shown in Fig. 2. Note that some adducts were occasionally removed during the fitting because of their very low intensity, which usually leads to a highly uncertain temporal behaviour. In addition, some compounds especially monomers exhibit characteristic of organic peroxide, but are likely generated due to in-source fragmentation of large molecules. Such compounds are identified with annotation and removed from the final list as they usually have relatively long RTs in the dimer range. For instance, as shown in Fig. 2A, we found $C_{10}H_{16}O_2$ that eluting at the same

RT=20.70 min of $C_{20}H_{32}O_5$, which is clearly a fragment of $C_{20}H_{32}O_5$ due to the in-source fragmentation. All compound ID are checked individually, which finally resulted in a list of organic peroxides (in this case 374 organic peroxides found in α-pinene SOA) containing information of their formula, RT, types of adduct, peroxide-iodide reactivity, as summarized in Table S4 and supplementary excel sheet Data S1. This step is achieved by a GUI module (see Fig. S2) to allow for data visualization and efficient filtering.

Text S2: Additional details of iodometry kinetic experiments for peroxide standards

In this study, all the iodometry kinetic experiments were performed in ACN-dominated solvent (95% ACN + 5% H_2O), which can minimize other unwanted processes such as the hydrolysis of peroxides. Normally, I_3^- absorption peaks were reported at 288 nm and 350-353 nm in water solvent.[2-4] However, it is unsure to what extent the I_3^- absorption spectra may change if switching from water to ACN-dominated solvent. Therefore, we performed iodometry in two solutions that both containing 45 μM H_2O_2, 20 mM KI and 30 mM acetic acid, but prepared in two different solvents (100% H_2O vs. 95% ACN + 5% H_2O). As shown in Fig. S3A, we observed a steady increase of I_3^- absorption in both solutions, and both of which reached to their maximum within one day, suggesting the completion of iodometry. Compared with pure water solvent, we observed a small peak shift from 351 nm to 361 nm when I_3^- was present in ACN-dominated solvent. We found an overlapping wavelength of 353 nm in both solutions, which was suggested to share the same I_3^- molar absorption coefficient. This is because both solutions should result in the same I_3^- concentration after the completion of iodometry, as the initial H_2O_2 concentrations were the same. Therefore, we used the absorbance value at 353 nm for I_3^- quantification in ACN-dominated solvent. The I_3^- molar absorption coefficient of 2.59×10^4 M^{-1} cm^{-1} at 353 nm [3] was adapted in this study, which is very close to other reported I_3^- molar absorption coefficients such as 2.32×10^4 M^{-1} cm^{-1} at 350 nm [4] and 2.645×10^4 M^{-1} cm^{-1} at 351 nm [2].

For the iodometry kinetic experiments with those 11 peroxide standards, their initial concentrations were estimated to be ~45 μM based on weighting and dilution, though there might have uncertainty in the stock solution due to their intrinsic labile property. In addition, PAA and 3-CA are known to be unstable and they are existed with H_2O_2 in equilibrium. 7 out of 11 peroxide standards were completely consumed in these iodometry kinetic experiments, which allows for quantification of final I_3^- concentration using the I_3^- molar absorption coefficient of 2.59×10^4 M^{-1} cm^{-1} at 353 nm. The peroxide consumption and I_3^- formation follow the stoichiometric ratio of 1:1 during iodometry, except 1:3 for 2-BP that has three -O-O- groups. As summarized in Table S3, the quantified final I_3^- concentrations agree fairly well with the initial peroxide concentration estimated to be ~45 μM, suggesting that the actual initial concentrations of these peroxide standards are comparable in iodometry kinetic experiments.

Since iodide was added in large excess in iodometry kinetic experiments, the peroxide-iodide reactivity (k_{1st}) for these peroxide standards can be determined as 1st order rate constant, which were either fitted or estimated according to below three categories:

(i) As shown in Fig. S3B, PAA and 3-CA are super-reactive peroxides and their iodometry reactions complete within 1 s upon the addition of KI, where our UV-Vis spectrometer was operated at fastest acquisition mode for measuring the absorbance at single wavelength of 350 nm. The e-folding lifetime ($\tau_{1/e}$) of PAA and 3-CA was determined to be 1 s as an upper limit, and therefore the corresponding k_{1st} value was estimated to be 8.64 × 10^4 d^{-1} as a lower limit.

(ii) For those peroxide standards such as BP, 2-BP, H_2O_2, CH and t-BP (see Fig. S3C-E), a steady increase of I_3^- until reaching its maximum was observed in their iodometry kinetic experiments. We used a function of y = A[1 - exp(-Bx)] to fit the I_3^- temporal trends, where A is actually the maximum concentration of I_3^-, and B is the k_{1st} value. Note that dissolved O_2 could very slowly oxidize I^- and lead the formation of I_3^-. To minimize such interference from O_2, the I_3^- temporal profiles for some peroxide standards with intermediate reactivity (2-BP, H_2O_2, CH and t-BH, see Fig. S3D-E) have been corrected by subtracting I_3^- from blank iodometry kinetic experiments without adding peroxide standard.

(iii) As shown in Fig. S3F, t-BPA, t-BPB, t-BP and DP are slowly reactive peroxides, which are not possible to reach full completion within several weeks in iodometry kinetic experiments. Therefore, their k_{1st} values cannot be fitted as those described in (ii). Alternatively, we calculate the decay profile of these peroxides by subtracting their quantified I_3^- concentration (the initial peroxide concentration X_0 is assumed as 45 μM), which allows us to fit their k_{1st} values as shown in Fig. S3G. Since we did not consider other unwanted processes for these four peroxides (e.g. O_2 might also slowly oxidize I^- and lead to I_3^- formation), the k_{1st} values obtained for those four peroxides were only estimated as an upper limit.

Table S1. Summary of parameters for LC-HRMS analyses in this study

LC parameters	
Analytical column	Waters HSS T3 UPLC column (100 mm × 2.1 mm, 1.8 μm)
Column temperature	40 °C
Injection volume	1-5 μL
Autosampler temperature	8 °C
Mobile phase - negative mode	A1: H_2O + 10 mM acetic acid; B: methanol
Mobile phase - positive mode	A2: H_2O + 0.1% formic acid; B: methanol
Flow rate	0.3 mL/min
Gradient - time (min)	% of B
0	0.1
1	0.1
26	99.9
28	99.9
30	0.1
HRMS parameters	
Ion source	heated electrospray ionization (H-ESI)
Polarity	negative and positive
Spray voltage	3.4 kV
Sheath gas flow	60 a.u.
auxiliary gas flow	15 a.u.
sweep gas flow	1 a.u.
capillary temperature	320 °C
auxiliary gas heater temperature	150 °C
scan mode	full MS mode
scan range	*m/z* 55 to 800
MS resolution	70,000 at *m/z* = 200
automated gain control (AGC) target	3.00E+06
maximum injection time	25 ms

Table S2. Composition of non-treated and KI-treated samples for the SOA iodometry kinetic experiments using LC-HRMS

	α-pinene SOA extract (in ACN) *	600 mM acetic acid (in ACN)	400 mM KI (in H_2O)	H_2O
Non-treated (200 μL)	180 μL	10 μL	0	10 μL
KI-treated (200 μL)	180 μL	10 μL	10 μL	0

*Note: α-pinene SOA extract was combining from 10 filter extracts (each 40 μL).

Table S3. Summary of final I_3^- concentration, pseudo-first-order rate constant (k_{1st}), and *e*-folding lifetime ($\tau_{1/e}$) obtained from the iodometry kinetic experiments for 11 peroxide standards (from Fig. S3).

Name	Final I_3^- (μM)	k_{1st} (d^{-1})	$\tau_{1/e}$
[1] peracetic acid (PAA)	30.2	$> 8.64 \times 10^4$	< 1 s
[2] 3-chloroperbenzoic acid (3-CA)	38.2	$> 8.64 \times 10^4$	< 1 s
[3] benzoyl peroxide (BP)	41.1	1.65×10^3	0.88 min
[4] 2-butanone peroxide (2-BP)	146.4	15.4 ± 1.5	1.6 h
[5] hydrogen peroxide (H_2O_2)	45.1	9.83 ± 0.12	2.4 h
[6] cumene hydroperoxide (CH)	53.4	0.82 ± 0.01	1.2 d
[7] *tert*-butyl hydroperoxide (t-BH)	53.4	0.26	3.8 d
[8] *tert*-butyl peroxybenzoate (t-BPB)	/	$< 4.56 \times 10^{-2}$	> 22 d
[9] *tert*-butyl peracetate (t-BPA)	/	$< 1.66 \times 10^{-2}$	> 60 d
[10] *tert*-butyl peroxide (t-BP)	/	$< 9.32 \times 10^{-3}$	> 107 d
[11] dicumyl peroxide (DP)	/	$< 4.36 \times 10^{-3}$	> 229 d

Table S4. List of top 50 organic peroxides (sorted by intensity) in α-pinene SOA. A complete list of 374 organic peroxides identified in α-pinene SOA is provided in an excel sheet as supplementary Data S1

rank #	comp. ID	predicted formula	RT (min)	intensity	intensity frac. (%)	k_{1st} (d^{-1})	$\tau_{1/e}$ (d)	primary adduct	other adducts found for merged fitting
1	9612	C20H32O5	20.70	5.4E+06	5.8	1.6E-01	6.28	[M+Na]+	[M+NH4]+, [M+H]+
2	8788	C16H26O5	19.86	5.2E+06	5.6	5.5E-02	18.08	[M+Na]+	[M+H]+, [M+K]+
3	9820	C20H32O5	20.97	5.0E+06	5.4	4.0E-02	25.11	[M+Na]+	[M+NH4]+, [M+K]+
4	1622	C10H16O5	9.34	2.7E+06	2.9	1.5E+01	0.07	[M+Na]+	[M+NH4]+, [M+H]+
5	2016	C10H18O4	10.08	2.0E+06	2.2	3.2E-02	31.45	[M+Na]+	[M+H]+
6	9128	C17H30O5	20.16	1.9E+06	2.1	3.5E-02	28.78	[M+Na]+	[M+NH4]+, [M+H-H2O]+
7	6547	C20H32O7	16.88	1.9E+06	2.0	8.2E-02	12.16	[M+Na]+	[M+NH4]+, [M+H]+
8	7264	C20H32O6	17.78	1.8E+06	2.0	1.2E+01	0.08	[M+Na]+	[M+NH4]+,[M+Na-H2O]+, [M+NH4-H2O]+
9	4998	C18H28O7	15.19	1.6E+06	1.8	2.4E-02	40.90	[M+Na]+	[M+NH4]+
10	2769	C10H18O5	11.09	1.6E+06	1.8	1.1E+01	0.09	[M+Na-H2O]+	[M+Na]+, [M+NH4-H2O]+
11	4830	C18H28O9	15.04	1.5E+06	1.7	1.8E-01	5.46	[M+H]+	[M+NH4]+, [M+H-H2O]+
12	4504	C17H26O7	14.68	1.5E+06	1.6	8.1E-02	12.39	[M+H]+	[M+NH4]+
13	5481	C16H26O7	15.83	1.4E+06	1.5	1.2E-01	8.55	[M+Na]+	[M+NH4]+, [M+H]+, [M+K]+, [M+H-H2O]+
14	1757	C8H12O5	9.52	1.4E+06	1.5	9.9E-02	10.13	[M+Na]+	[M+NH4]+, [M+H-H2O]+
15	3900	C12H20O5	13.57	1.3E+06	1.4	2.1E+01	0.05	[M+Na]+	[M+K]+, [M+NH4-H2O]+
16	8933	C34H50O10	19.90	9.1E+05	1.0	8.8E-02	11.38	[M+H]+	
17	9057	C19H30O5	20.11	7.9E+05	0.8	7.9E-02	12.58	[M+Na]+	
18	7195	C21H34O7	17.70	7.7E+05	0.8	9.1E-01	1.10	[M+Na]+	[M+NH4]+
19	6322	C20H32O9	16.59	7.5E+05	0.8	4.6E-01	2.19	[M+Na]+	
20	1697	C11H20O5	9.50	7.5E+05	0.8	9.6E+00	0.10	[M+Na]+	
21	2620	C9H16O3	10.76	7.2E+05	0.8	8.7E-02	11.43	[M+Na]+	
22	10445	C20H32O6	21.97	7.2E+05	0.8	8.5E-02	11.83	[M+Na]+	[M+Na-H2O]+, [M+NH4-H2O]+
23	6254	C20H32O8	16.48	6.7E+05	0.7	1.1E+00	0.87	[M+Na]+	[M+NH4]+
24	5140	C10H18O3	15.34	6.3E+05	0.7	3.3E+00	0.30	[M+Na]+	[M+NH4]+
25	9226	C19H30O5	20.23	6.3E+05	0.7	3.9E-02	25.71	[M+Na]+	[M+NH4]+
26	1576	C9H16O6	9.31	6.0E+05	0.6	1.6E-01	6.31	[M+Na]+	
27	6530	C17H28O7	16.84	6.0E+05	0.6	9.5E-02	10.56	[M+Na]+	[M+NH4]+
28	2030	C11H20O6	10.13	5.9E+05	0.6	3.9E-01	2.58	[M+Na]+	
29	5322	C19H30O7	15.60	5.9E+05	0.6	1.7E-01	6.01	[M+Na]+	[M+NH4]+, [M+H]+
30	10276	C20H32O6	21.73	5.8E+05	0.6	2.2E-01	4.60	[M+Na]+	[M+NH4]+, [M+H]+
31	10411	C27H44O9	21.97	5.7E+05	0.6	1.3E+00	0.77	[M+Na]+	[M+NH4]+
32	1674	C9H14O4	9.34	5.7E+05	0.6	4.0E-01	2.47	[M+H]+	
33	4959	C19H32O9	15.15	5.7E+05	0.6	1.8E-01	5.54	[M+Na]+	[M+H-H2O]+
34	7629	C22H36O7	18.12	5.6E+05	0.6	6.1E-01	1.64	[M+Na]+	
35	3854	C16H26O8	13.45	5.3E+05	0.6	4.1E-01	2.43	[M+Na]+	[M+NH4]+
36	8681	C20H30O6	19.71	5.1E+05	0.6	1.8E-01	5.43	[M+Na]+	[M+NH4]+, [M+H]+
37	7936	C20H30O5	18.60	5.1E+05	0.5	1.7E-01	5.83	[M+Na]+	[M+NH4]+
38	7145	C19H30O6	17.65	5.1E+05	0.5	1.2E-01	8.07	[M+Na]+	[M+NH4]+, [M+H]+
39	5029	C17H28O6	15.20	5.0E+05	0.5	1.2E-01	8.46	[M+Na]+	[M+H]+
40	7046	C21H34O7	17.54	5.0E+05	0.5	7.8E-01	1.28	[M+Na]+	[M+NH4]+
41	9169	C16H26O4	20.20	5.0E+05	0.5	4.9E-02	20.37	[M+Na]+	[M+NH4]+
42	4668	C19H30O8	14.79	4.9E+05	0.5	1.9E+00	0.53	[M+Na]+	
43	8233	C19H30O7	18.88	4.8E+05	0.5	4.2E-01	2.37	[M+NH4]+	[M+Na]+, [M+K]+
44	6425	C19H30O7	16.70	4.7E+05	0.5	4.8E+01	0.02	[M+Na]+	[M+NH4]+
45	5905	C19H30O7	16.15	4.7E+05	0.5	4.1E-01	2.47	[M+Na]+	
46	9061	C20H32O6	20.11	4.5E+05	0.5	8.8E-01	1.14	[M+Na]+	[M+H]+
47	100411	C10H18O6	6.56	4.4E+05	0.5	1.0E+01	0.10	[M+Na]+	[M+NH4]+
48	8970	C19H30O5	19.96	4.3E+05	0.5	3.0E-01	3.35	[M+NH4]+	
49	8990	C20H32O6	19.99	4.2E+05	0.4	4.8E-01	2.07	[M+Na]+	[M+H]+
50	6463	C20H32O8	16.75	4.1E+05	0.4	9.2E-01	1.09	[M+Na]+	[M+NH4]+

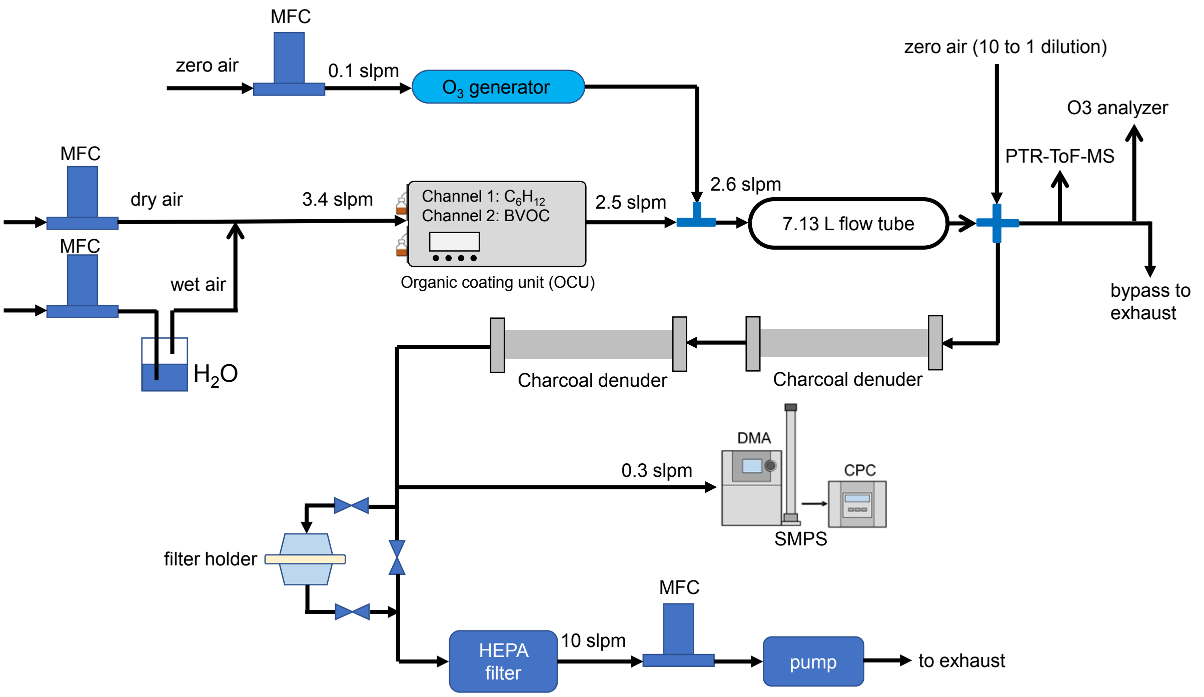

Fig. S1 Laboratory flow tube setup for generation and collection of α-pinene SOA.

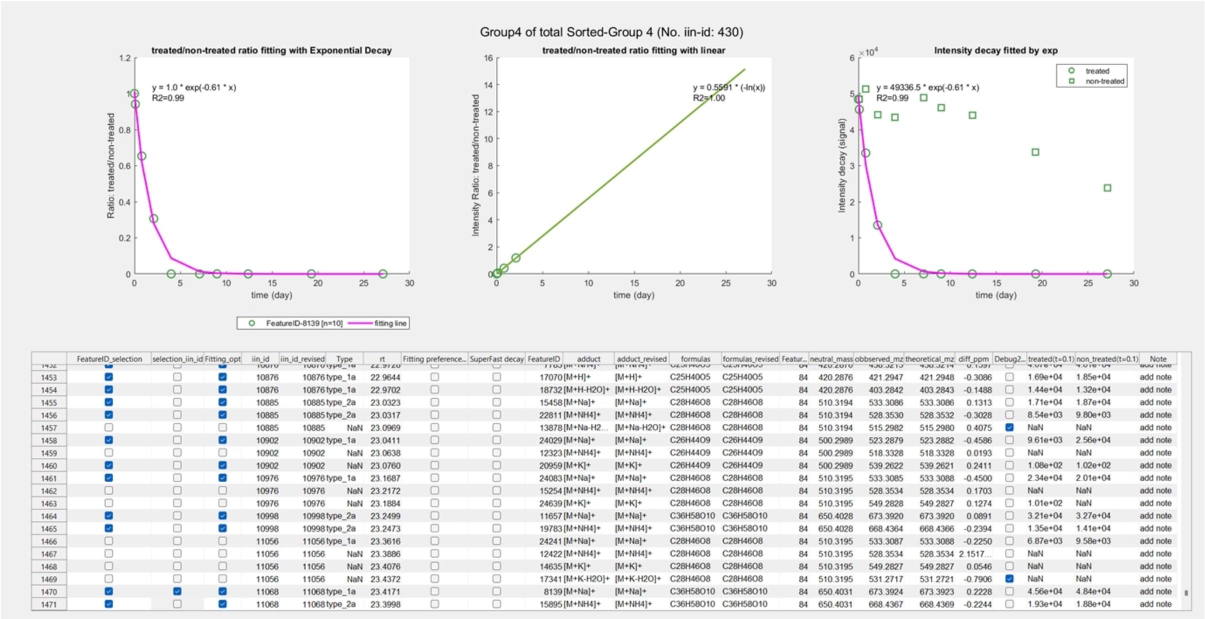

Fig. S2. Screenshot of the in-house data processing tool (with a GUI module), which allows for data visualization and efficient identification of individual organic peroxide in SOA based on the results from time-dependent iodometry-assisted LC-HRMS. The non-targeted analysis of LC-HRMS dataset was made from MZmine software.

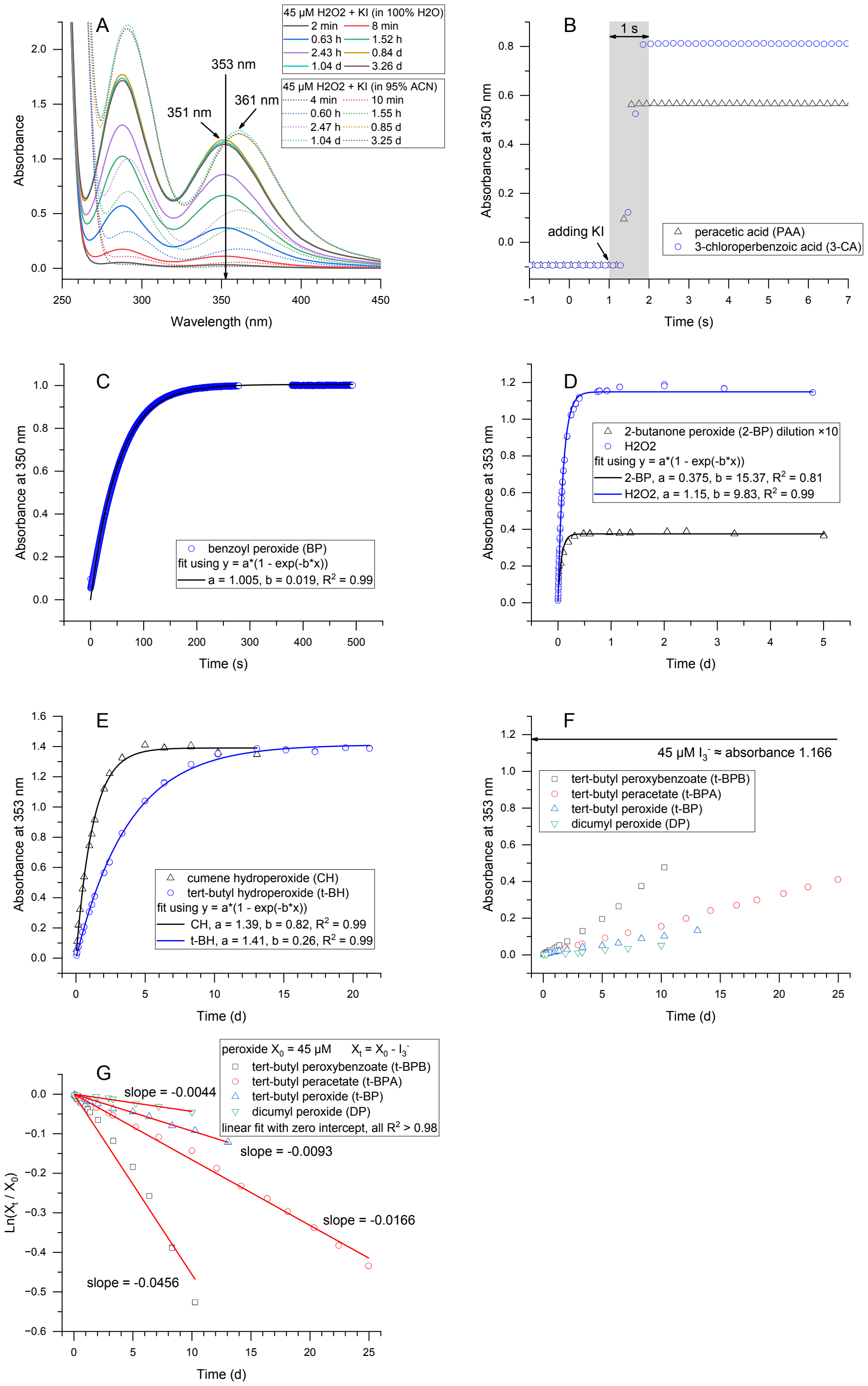

Fig. S3. UV-Vis measurements from iodometry kinetic experiments for 11 peroxide standards. (A) I_3^- absorption spectra from 45 μM H_2O_2 + 20 mM KI + 30 mM acetic acid in 100% H_2O vs. ACN-dominated solvent (95% ACN + 5% H_2O); (B-F) temporal profiles of I_3^- absorbance at 350 or 353 nm; (G) natural log normalized peroxide decays derived from (F) by assuming the initial peroxide concentration of 45 μM.

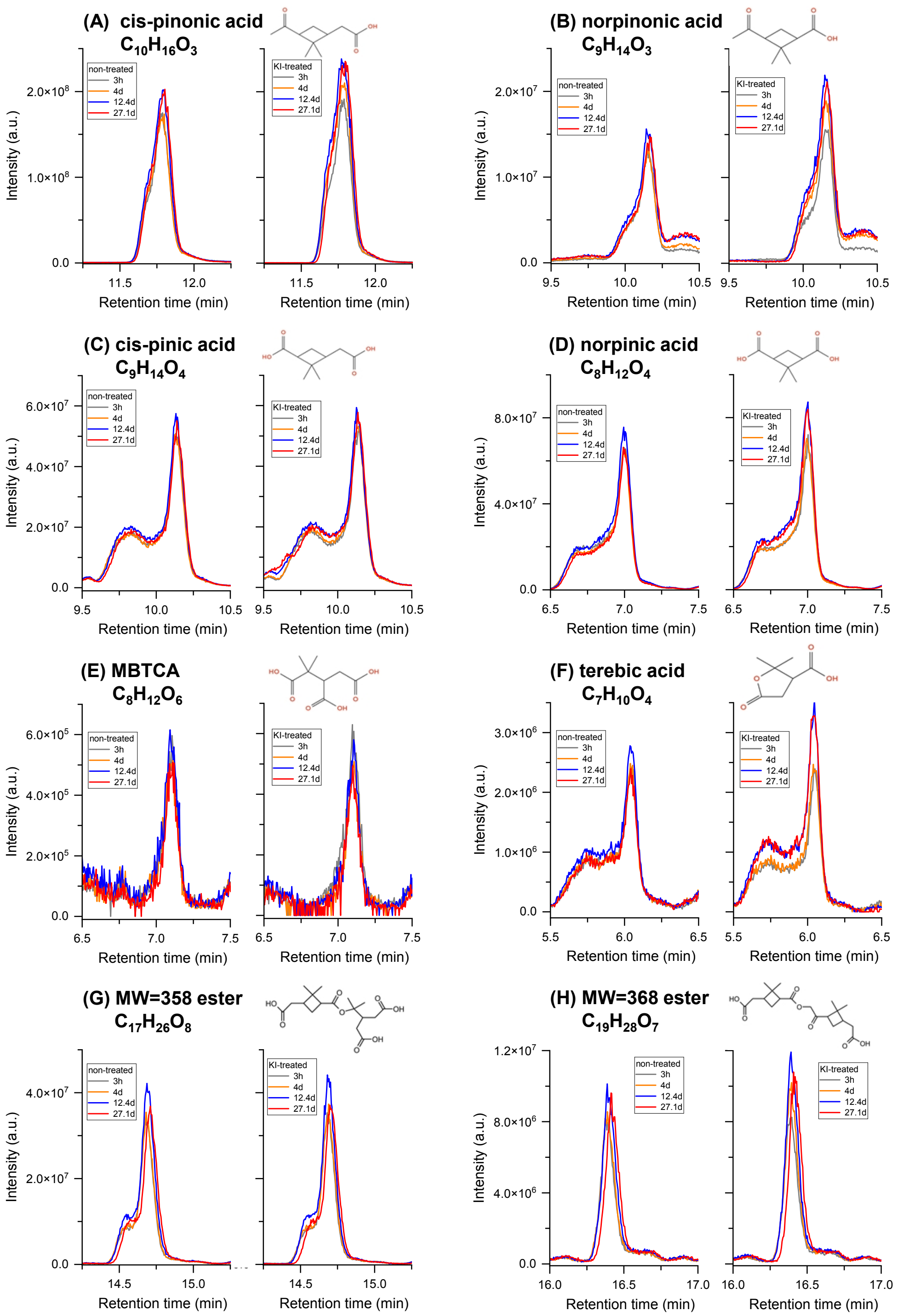

Fig. S4. Time-dependent extracted ion chromatograms (EICs) of $[M+Na]^+$ for various non-peroxides from KI-treated and non-treated dataset, which have been well characterized in α-pinene SOA in previous studies. [5-8] Cis-pinonic acid (A), cis-pinic acid (C), MBTCA (E) and terebic acid (F) were identified with reference standards, while norpinonic acid (B), norpinic acid (D), MW=358 ester (G) and MW=368 ester (H) were identified with high confidence.

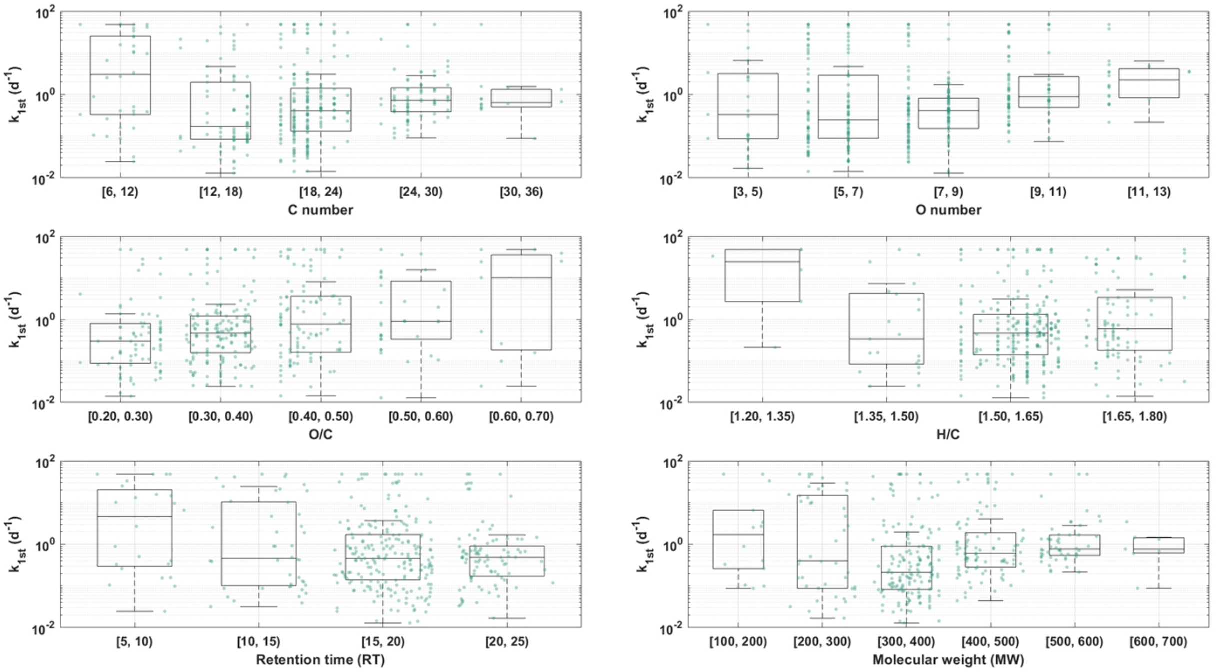

Fig. S5. Correlation between peroxide-iodide reactivity (k_{1st}) and their molecular characteristics from 374 organic peroxides. The x axis of scatters is grouped into different bins and presented as box plots (10th, 25th, 50th, 75th and 90th).

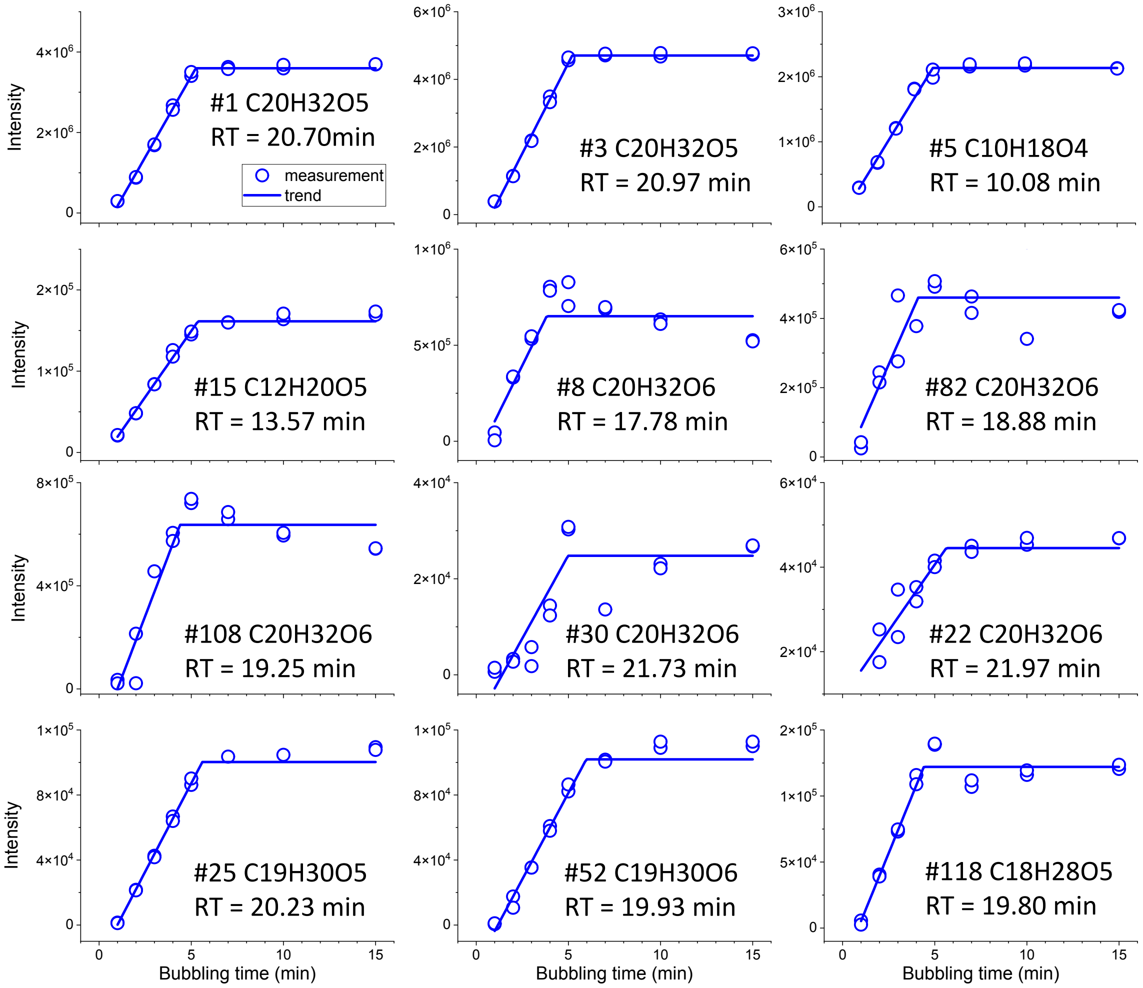

Fig. S6. Time series of 12 SCI-derived organic peroxides identified during the liquid-phase ozonolysis of α-pinene (rank # refers to Table 1).

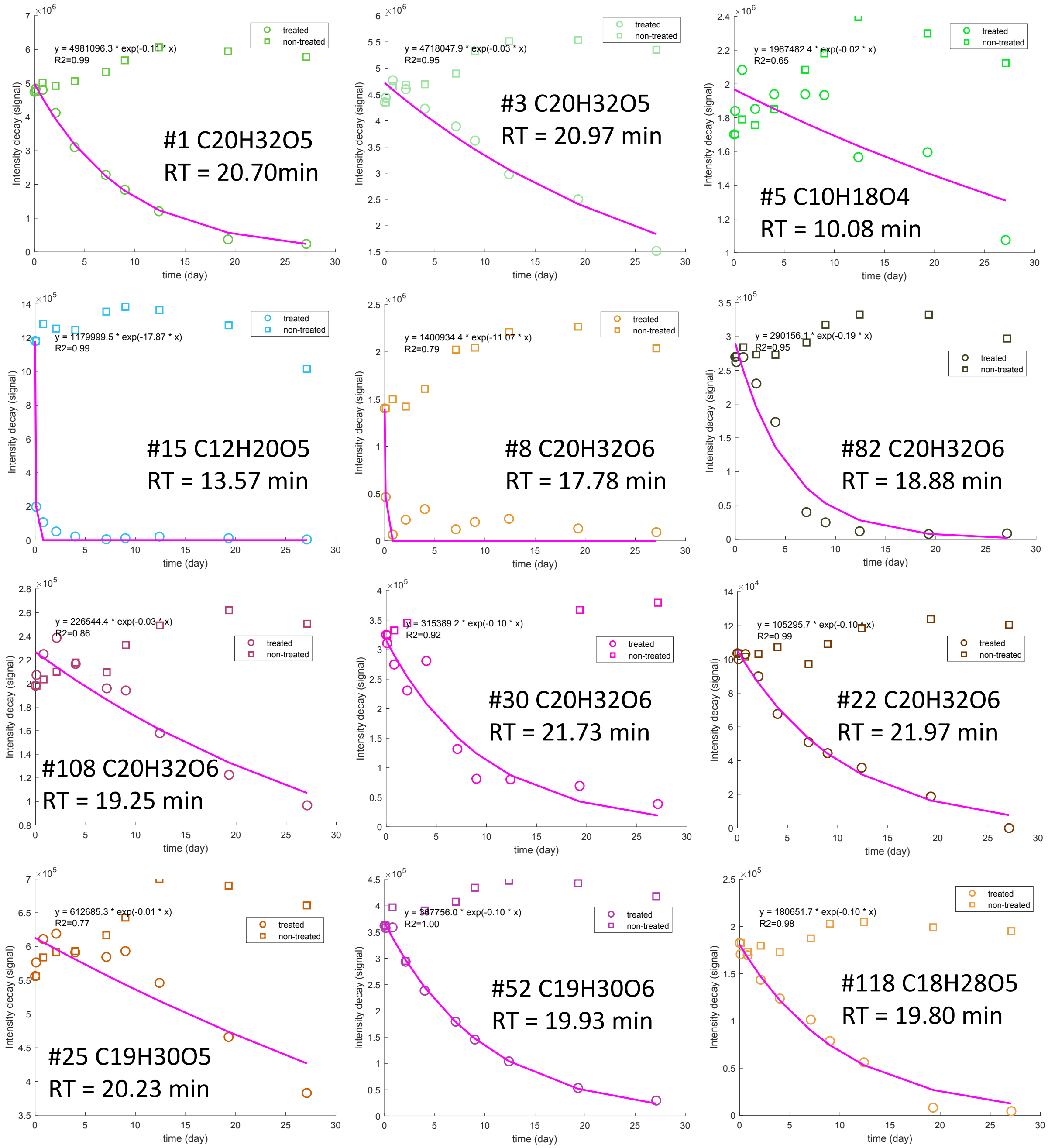

Fig. S7. Time series of 12 SCI-derived organic peroxides (rank # refers to Table 1) in α-pinene SOA in KI-treated (circles) and non-treated (squares) dataset, as measured in SOA iodometry kinetic experiments over four weeks.

References

(1) Schymanski, E. L.; Jeon, J.; Gulde, R.; Fenner, K.; Ruff, M.; Singer, H. P.; Hollender, J. Identifying small molecules via high resolution mass spectrometry: communicating confidence. *Environ Sci Technol* **2014**, *48* (4), 2097-2098. DOI: 10.1021/es5002105.

(2) Klassen, N. V.; Marchington, D.; McGowan, H. C. E. H2O2 Determination by the I3- Method and by KMnO4 Titration. *Analytical Chemistry* **1994**, *66* (18), 2921-2925. DOI: 10.1021/ac00090a020.

(3) Fournier, M. C.; Falk, L.; Villermaux, J. A new parallel competing reaction system for assessing micromixing efficiency—Experimental approach. *Chemical Engineering Science* **1996**, *51* (22), 5053-5064. DOI: 10.1016/0009-2509(96)00270-9.

(4) Wei, Y. J.; Liu, C. G.; Mo, L. P. Ultraviolet absorption spectra of iodine, iodide ion and triiodide ion. *Guang Pu Xue Yu Guang Pu Fen Xi* **2005**, *25* (1), 86-88.

(5) Yu, J.; Cocker Iii, D. R.; Griffin, R. J.; Flagan, R. C.; Seinfeld, J. H. Gas-Phase Ozone Oxidation ofMonoterpenes: Gaseous and Particulate Products. *Journal of Atmospheric Chemistry* **1999**, *34* (2), 207-258. DOI: 10.1023/a:1006254930583.

(6) Ma, Y.; Luciani, T.; Porter, R. A.; Russell, A. T.; Johnson, D.; Marston, G. Organic acid formation in the gas-phase ozonolysis of alpha-pinene. *Phys Chem Chem Phys* **2007**, *9* (37), 5084-5087. DOI: 10.1039/b709880d.

(7) Yasmeen, F.; Vermeylen, R.; Maurin, N.; Perraudin, E.; Doussin, J.-F.; Claeys, M. Characterisation of tracers for aging of α-pinene secondary organic aerosol using liquid chromatography/negative ion electrospray ionisation mass spectrometry. *Environmental Chemistry* **2012**, *9* (3). DOI: 10.1071/en11148.

(8) Kahnt, A.; Vermeylen, R.; Iinuma, Y.; Safi Shalamzari, M.; Maenhaut, W.; Claeys, M. High-molecular-weight esters in α-pinene ozonolysis secondary organic aerosol: structural characterization and mechanistic proposal for their formation from highly oxygenated molecules. *Atmospheric Chemistry and Physics* **2018**, *18* (11), 8453-8467. DOI: 10.5194/acp-18-8453-2018.
